# Association of Rotating Night Shift Work with Body Fat Percentage and Fat Mass Index among Female Steelworkers in North China

**DOI:** 10.3390/ijerph18126355

**Published:** 2021-06-11

**Authors:** Shengkui Zhang, Han Wang, Yongbin Wang, Miao Yu, Juxiang Yuan

**Affiliations:** 1 Department of Epidemiology and Health Statistics, School of Public Health, North China University of Science and Technology, Tangshan 063210, China; zhangsk@stu.ncst.edu.cn (S.Z.); wanghan@stu.ncst.edu.cn (H.W.); yumiao@stu.ncst.edu.cn (M.Y.); 2 Department of Epidemiology and Health Statistics, School of Public Health, Xinxiang Medical University, Xinxiang 453003, China; 191035@xxmu.edu.cn

**Keywords:** night shift work, body mass index, obesity

## Abstract

The aim of this study was to evaluate the associations of rotating night shift work with body fat percentage (BF%) and fat mass index (FMI). A cross-sectional study was conducted among 435 female steelworkers, aged 26–57 years in Tangshan, China. BF% was assessed via bioelectrical impedance analysis and FMI was calculated. Different exposure metrics of night shift work were used to examine the effects of night shift work on BF% and FMI. The duration (years), cumulative number (nights), and cumulative length of night shifts (hours) were positively correlated with FMI and BF%, and these relationships were independent of body mass index (BMI). Compared with day workers, night shift workers with an average frequency of night shifts >7 nights/month (odds ratio (OR) 2.50, 95% confidence interval (CI) 1.17 to 5.35) and percentage of hours on night shifts >30% (OR 2.55, 95% CI 1.21 to 5.39) had elevated odds of obesity (BF% ≥ 35.0%). Nonobese night shift workers by the BMI criterion should also be alert to the risk of the excess accumulation of body fat, which is actually responsible for most obesity-associated adverse health consequences. Health interventions for related populations need to be improved, which is currently more focused on overall weight control.

## 1. Introduction

Night shift work is a method of the organization of working time, in which different staff members or groups succeed one another at the workplace during the regular sleeping hours of the general population. Our round-the-clock society is dependent on shift work, despite a growing body of evidence that has shown that the misalignment of circadian rhythms can have a series of pronounced adverse effects on health. Shift work has been identified as an important occupational hazard, affecting about 20% of workers worldwide [1]. Evidence is particularly extensive with regard to the link between night shift work and obesity [2,3].

Obesity is an important risk factor contributing to the overall burden of disease worldwide [4]. In China, the prevalence of obese or overweight was about 15% for children and 46% for adults [5]. What we already know is that obesity has been identified as an independent risk factor for cardiovascular disease (CVD) [6]. It should be noted that body mass index (BMI) is the most commonly used proxy for assessing overall body fatness and the level of obesity, as it is easy to measure and inexpensive [7]. However, the limitation of BMI is that it cannot distinguish between body fat mass and lean mass. For example, muscular athletes will also have a higher BMI without excessive accumulation of body fat. In fact, it is the excess accumulation of body fat that is responsible for most obesity-associated adverse health outcomes [6]. This finding lends support to the idea that BMI classification may underestimate the risk of CVD for subjects with elevated adiposity, while the body composition index (body fat percentage, BF%) may overcome this deficiency [8]. Moreover, previous studies have identified high fat mass index (FMI, the fat mass in kilograms divided by the square of the height in meters) as a good proxy for metabolic syndrome and cardiovascular risk [9,10,11]. Similar to BMI, FMI can eliminate the effect of height on BF% after body size is normalized, so it can describe the distribution of body fat, which is closely related to the risk of CVD [12]. A growing body of evidence suggests that shift work has been associated with CVD [13,14]. However, the relationship between shift work and increased BMI is inconsistent. Meanwhile, whether the duration, frequency, and intensity of night shift work are related to FMI and BF%, which may be more closely associated with CVD, is currently inconclusive.

A previous study showed that night shift workers had a greater BF% than day workers within the same BMI range, and it suggested that this could be associated with the disruption of appetite-regulating hormones from the upper gut [15]. In addition, the association between body composition (a combined index including both BMI and BF%) and lifetime shift work exposure has been evaluated [16]. Moreover, there is an increasing body of evidence suggesting an association between night shift work and abdominal obesity [2,17]. However, evidence regarding the independent effects of important exposure characteristics of shift work on BF% and FMI remains sparse.

Therefore, understanding the association of different exposure characteristics of shift work with body fat and its distribution is important for the formulation of effective strategies for prevention that are currently more focused on overall weight control. To the best of our knowledge, there is currently no study on the relationship between different exposure characteristics of shift work and body composition. In the present study, different exposure metrics of night shift work, including current night shift status, duration of night shifts (years), cumulative number of night shifts (nights), cumulative length of night shifts (hours), average frequency of night shifts (nights/month), and percentage of hours on night shifts, were used to examine the effects of rotating night shift work on FMI and BF% (based on the bioelectrical impedance analysis method) among female steelworkers.

## 2. Materials and Methods

### 2.1. Study Design and Population

This was a cross-sectional study conducted at 11 steel production departments owned by the HBIS Group’s Tangsteel Company in Tangshan City, Hebei Province in north China. The study design and population have been described in detail in our previous studies [18,19,20]. Given that a previous study has evaluated the association between night shift work and obesity among male steelworkers in this population, the present study only included female workers [21]. All subjects, enrolled between February and June 2017, were required to complete anthropometric measurements and a detailed questionnaire by face-to-face interview. A total of 631 female workers at this company underwent the legally required annual health examination in 2017, of which 586 people participated in this study. Dust, heat stress, noise, and carbon monoxide are the major occupational hazards to the current workers [19]. Workers without detailed lifetime shift work information, those who provided incomplete covariate data on the questionnaire, and those who did not complete the anthropometric measurements were excluded. Finally, 435 female steelworkers were included. The sample size estimation was completed by the procedure “Tests for Two Proportions (Odds Ratios)” of Power Analysis and Sample Size (PASS). Based on the characteristics of the present participants, when the power (1-Beta) was set to 0.80, the alpha (significance level) was set to 0.05, the odds ratio (OR) was set to 2.5, the P2 (the proportion of obesity (BF% ≥ 35.0%) among day workers) was set to 0.121, the R (sample allocation ratio = N2/N1) was set to 3, and the estimated N1 and N2 were 82 and 246, respectively. N1 (night shift workers) and N2 (day workers) are the sizes of the samples drawn from the corresponding populations. This research was approved by the Ethics Committee of North China University of Science and Technology (No. 16040). All participants gave informed consent before taking part in this study.

### 2.2. Anthropometric Measurements

The values of weight, fat mass, and BF% were measured by the Body Composition Analyzer (TANITA BC-420, Japan). Before the measurement, all the participants were required to remove keys, jewelry, and other metal products, and were in a state of fasting and emptying their bladders in the morning. In order to minimize measurement error, the body composition analyzer was calibrated every day before measuring. After entering the basic information, the participants should remove heavy coats, wear only light clothing, keep their feet bare, and stand upright on the electrode to complete the measurement. A detailed description of the body composition analyzer of the same brand and model can be found in the China national health survey (CNHS) [22]. First, 20 participants (5%) were randomly selected and their BF% was measured again by bioelectrical impedance analysis (BIA) and dual-energy X-ray absorptiometry (DXA). The intra-rater reliability of the measurements (BIA method) was 98.3% (*p* < 0.001). Lin’s concordance correlation coefficient (LCCC) of the two methods was 0.92 (95%CI: 0.81 to 0.97) with a standard error of 0.04. The height was measured by an ultrasonic height-weight instrument (DK, 08-C, Beijing). Before the measurement, participants should remove all hats and other headwear. Participants should then stand close to the column of the instrument with their heels together and their forefeet 60° apart with their toes splayed and their arms hanging down naturally. They should make sure the head, shoulder, hips, and heels are close to the instrument. They should keep the ear canals and lower orbit in the same horizontal position. The participants should stand as straight as possible and breathe deeply [22]. The height data that were ultimately used for analysis were accurate to 0.1 cm. The BMI was defined as the body weight in kilograms (kg) divided by the square of the body height in meters (m^2^). The FMI was defined as the fat mass (kg) divided by the square of the body height (m^2^). The waist-to-hip ratio (WHR) was calculated by dividing the waist circumference (WC) by hip circumference (HC). The waist-to-height ratio (WHtR) was calculated by dividing the waist circumference (WC) by height. BMI ≥ 28 kg/m^2^ (for Chinese adults) [23] was defined as obesity. Based on the most frequently used cutoff points in the literature, BF% ≥ 35.0% was used for defining obesity for women [8].

### 2.3. Assessment of Night Shift Work

The main work schedule of the present study population has been introduced in detail in our previous research [19,20]. In brief, shift work in this study refers to rotating night shifts (the mainly four-crew-three-shift system now and historical three-crew-two-shift system). Workers who worked regular working hours were defined as day workers. In this study, the detailed lifetime employment history was collected by face-to-face personal interviews and all the reported information was verified with the company’s records. Participants who were recruited were asked to report whether they were involved in rotating night shift work (working through 00:00 to 6:00) during their employment [24]. If yes, they would be further asked about the start and end dates of each shift system, the average number of night shifts per month in each shift system, hours spent in each different night shift system (hours/night), and usual days off per month. Using the above work schedule information, the duration of night shift work (years) (sum of years spent in all different night shift systems), cumulative number of night shifts (nights) (sum of nights spent in all different night shift systems), cumulative length of night shifts (hours) (sum of hours spent in all different night shift systems), average frequency of night shifts (nights/month), and percentage of hours on night shifts (%) (cumulative length of night shifts divided by total working hours of employment) were aggregated.

### 2.4. Assessment of Covariates

A structured questionnaire was used after repeated revisions. All information in the questionnaire was collected through face-to-face surveys. After checking the completeness and correctness of each questionnaire, we used a customized program to scan and transformed the handwritten data into an electronic data set immediately. The transformed version of each questionnaire was checked by two skilled investigators separately, and then the checked results were compared and reviewed before the final submission. The questionnaire mainly includes age, ethnicity, work schedule, smoking, drinking, educational level, physical activity, sleep duration, and insomnia. Smoking and drinking status were evaluated from self-reported information and were divided into “never,” “previous,” and “current.” Only 11 (2.5%) participants were ever smokers, so we combined “previous” and “current” smoker into “Pre-/Current smoker.” Only 11 (2.5%) participants were ever drinkers, so we combined “previous” and “current” drinker into “Pre-/Current drinker.” Marital status was divided into “single,” “married/cohabitating,” and “divorced/widow.” Only 4 (0.9%) participants reported as single, so we combined “single” into the “Single/Divorced/Widow” group. The level of education was divided into two categories: “High school or below,” and “university or college.” Dietary intake information was collected by a validated semiquantitative food frequency questionnaire [25]. Participants were asked to report the average frequency they had consumed on each food of a standard portion size over the previous 12 months. Diet quality scores were assessed based on the DASH diet score [26]. The calculation of metabolic equivalents was based on the International Physical Activity Questionnaire (IPAQ) [27]. The assessment of insomnia was estimated using the Athens Insomnia Scale (AIS), and AIS score ≥ 6 was defined as insomnia [28]. Sedentary behavior (hours/day) was assessed using a set of open-ended questions on the average working days and rest days time spent over the last four weeks on: television viewing (including DVDs and videos) and any other sitting during leisure time (including reading, studying, using a computer, and playing video games) [29]. The durations of sleep and sedentary behavior were the weighted averages of sleep and sedentary behavior on working days and rest days, respectively. Exposure to LAN was assessed through participants’ reports about the brightness of the bedroom ambient at night. Participants were asked to class the brightness of their bedroom LAN into the following four categories: “you wear a mask or too dark to see your fingers”; “light enough to see your fingers but not to identify the indoor environment clearly”; “light enough to identify the indoor environment clearly but not enough to read”; “light enough to read”. The two lightest categories were combined because of small numbers in the lightest category (2.41%). Finally, brightness of the bedroom ambient at night was divided into three categories: “darkest,” “middle,” and “lightest.” The participants were also asked to report the usual number of times a light was on per night when the subject’s sleep was interrupted, and the living duration of the current residence [30,31]. The assessment of the current use of oral contraceptives (yes/no) and menopausal status (premenopausal/postmenopausal) relied on self-reported information.

All related occupational hazard factors were measured by a qualified third-party company in accordance with the National Occupational Health Standards of the People’s Republic of China (the major occupational hazards including dust, heat stress, noise, and carbon monoxide) (see Supplementary Materials).

### 2.5. Statistical Analysis

Continuous variables are presented as means and standard deviations (SD), and between-group comparisons were performed using Student’s *t*-test if the data were normally distributed. Otherwise, the median (upper quartile-lower quartile) and Wilcoxon Scores (Rank Sums) test were used to describe and compare these continuous variables between groups. The partial Spearman correlation coefficients were used to assess the relationship between body composition indices (BF%, FMI, BMI, WC, WHR, and WHtR) and different exposure metrics of night shift work. Categorical variables are presented as numbers and percentages, and the chi-square test was used to compare differences between groups.

Multivariate logistic regression models were used to examine the relationships between night shift work and obesity (according to BMI and BF%, respectively). The risk factors of obesity reported in the literature (age, sleep duration, sleep quality, sedentary behavior, diet, physical activity, LAN, marital status, menopausal status, current use of oral contraceptives, and socioeconomic status) [17,26,30] and the potential confounders with an unbalanced distribution between shift and day-workers were included in the multivariate analysis. Generalized linear models (GLM) were used to assess the association between different exposure metrics of night shift work and anthropometric measures (BF%, FMI, BMI) with each anthropometric measure as the dependent variable, and different exposure metrics of night shift work and covariates described above as the independent variables using the Statistical Analysis System (SAS) procedure “PROC GENMOD.”

Restricted cubic spline (RCS) models were utilized to visually examine the association of the duration of night shifts (continuous), cumulative number of night shifts (continuous), and cumulative length of night shifts (continuous) with BMI, FMI, and BF% with adjustment for potential confounders.

Subsequently, potential mediators of the association between the duration of night shifts (ordered variable: 0, day work; 1, 1–13 years; 2, 14–20 years; 3, 21–26 years; 4, 27–38 years) and BMI, FMI, and BF% (continuous variable), including sleep duration (continuous variable), Athens Insomnia Scale (AIS) score (continuous variable, range 0 to 24), DASH score (continuous variable, 8 to 40), and physical activity (continuous variable, MET-h/week), were introduced to estimate the average causal mediation effects (ACME) using R package “mediation” (version 4.5.0) [32]. *p* < 0.05 was regarded as significant for 2-sided tests.

## 3. Results

### 3.1. General Characteristics of the Participants

General characteristics of the included and excluded participants were well balanced except for age, smoking, and drinking status (Table S1). Compared with workers excluded, those who were included were younger (44.1 ± 5.0 years vs. 45.1 ± 4.7 years, *p* = 0.020) and had a lower proportion of smoking (10.3% vs. 21.9%, *p* < 0.001) and drinking (8.3% vs. 17.9%, *p* < 0.001). The general characteristics of study participants according to shift status are presented in Table 1. The present study of 435 female workers consisted of 79.1% current or previous night shift workers, with a mean age of 44.1 years and a mean BMI of 23.8 kg/m^2^. In addition, 8.7% of participants reported the lightest brightness of bedroom ambient LAN exposure. Previous or current smoking and Han nationality were more likely to be reported among night shift workers. Compared with day workers, night shift workers had a higher DASH score, sedentary behavior hours, FMI, and BF%, but less sleep duration.

### 3.2. Different Exposure Metrics of Night Shift Work and Anthropometric Measures

**Figure 1:**
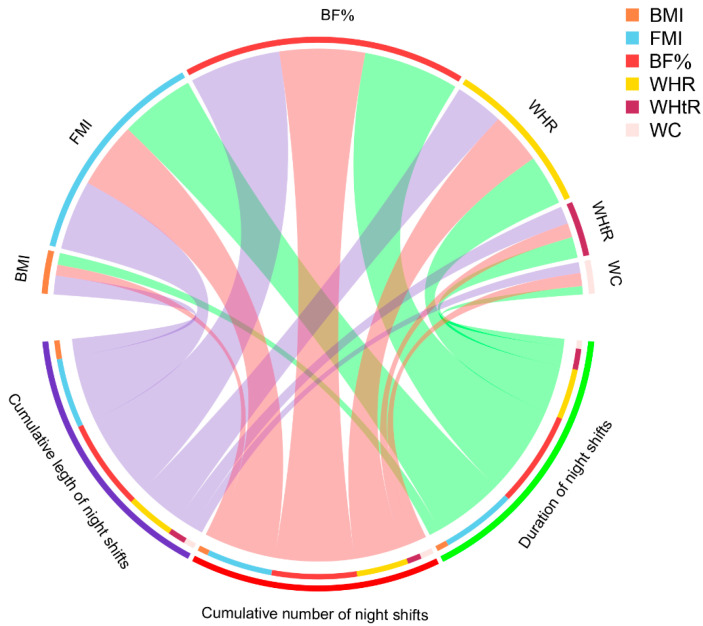
Chord diagram for partial correlations of different exposure metrics of night shift work with FMI and BF%. The width of the arcs in different colors represents the magnitude of the partial Spearman correlation coefficient. BMI, body mass index; BF%, body fat percentage; FMI, fat mass index; WC, waist circumference; HC, hip circumference; WHtR, waist-to-height ratio; WHR, waist-to-height ratio.

As shown in Figure 1, after adjusting for potential confounders, the correlations of different exposure metrics of night shift work (continuous) with FMI and BF% were closer than those with BMI and other indicators of abdominal obesity (the width of the arcs in different colors represents the magnitude of the partial Spearman correlation coefficient). In Table S2, we also show the values of the partial Spearman correlation coefficients between different exposure metrics of night shift work and anthropometric measures and p values for the significance test.

**Figure 2:**
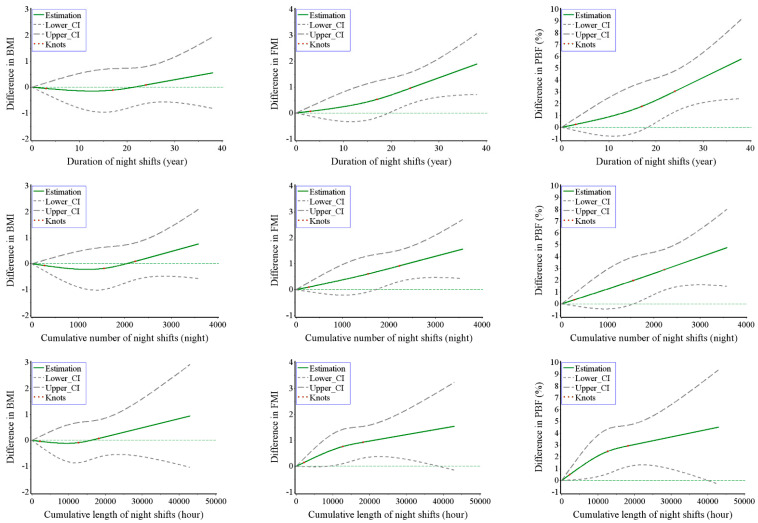
Associations of duration of night shifts (continuous), cumulative number of night shifts (continuous), and cumulative length of night shifts (continuous) with BMI, FMI, and BF%. The solid green lines, the long gray dashes, and the short gray dashes represent the point estimate of the difference and the upper and lower limits of its 95% CI, respectively. Knots are represented by red dots. “Difference in BMI (FMI or BF%),” represents the difference in BMI (FMI or BF%) between night shift workers with any value of duration of night shifts (cumulative number or cumulative length of night shifts) with day workers. Adjusted for age, smoking status, drinking status, education level, ethnicity, bedroom ambient light level, sleep duration, insomnia, sedentary behavior, DASH score, physical activity, marital status, menopausal status, and current use of oral contraceptives. BMI, body mass index; BF%, body fat percentage; FMI, fat mass index; CI, confidence interval.

Different exposure metrics of night shift work did not show significant associations with the continuous variable of BMI (Table 2). The GLM analysis revealed positive and significant associations of the duration of night shifts, cumulative number of night shifts, cumulative length of night shifts, average frequency of night shifts, and percentage of hours on night shifts with FMI and BF% (*p* trend < 0.05). Compared with day workers, the FMI increased by 1.028, 0.928, 0.961, 0.862, and 0.916 kg/m^2^, respectively, and BF% increased by 3.7%, 3.4%, 3.1%, 2.3%, and 2.5% respectively, for night shift workers in the highest exposure categories of duration of night shifts, cumulative number of night shifts, cumulative length of night shifts, average frequency of night shifts, and percentage of hours on night shifts. In the RCS models, positive correlations of FMI and BF% with duration of night shifts (continuous, years), cumulative number of night shifts (continuous, nights), and cumulative length of night shifts (continuous, hours) were observed (Figure 2).

No significant association between different exposure metrics of night shift work and obesity defined by BMI (≥28 kg/m^2^) was observed, regardless of whether the potential confounders were adjusted (Table 3). Compared with day workers, significantly elevated odds of obesity defined by BF% (≥35%) were observed in the highest exposure categories of night shift work (crude OR). After adjustment for age, smoking status, drinking status, education level, ethnicity, bedroom ambient light level, sleep duration, insomnia, sedentary behavior, DASH score, physical activity, marital status, menopausal status, and current use of oral contraceptives, these estimates were attenuated but remained significant (adjusted OR), with ORs (95% CI) of 3.48 (1.50–8.08), 3.11 (1.33–7.27), 3.35 (1.43–7.81), 2.50 (1.17–5.35), and 2.55 (1.21–5.39), respectively, for participants in the highest exposure categories of duration of night shifts, cumulative number of night shifts, cumulative length of night shifts, average frequency of night shifts, and percentage of hours on night shifts when compared with day workers.

### 3.3. Sensitivity Analyses

Given that poor sleep quality, short sleep duration, unhealthy dietary habits, and low physical activity may result in obesity, they could also be direct consequences of the exposure to night shift work, thus making these factors potential mediators of the association between the exposure to night shift work and risk of obesity. After adjustment for age, smoking status, drinking status, education level, ethnicity, bedroom ambient light level, sedentary behavior, marital status, menopausal status, and current use of oral contraceptives, the associations of duration of night shifts (ordered) with BMI (continuous), FMI (continuous), and BF% (continuous) did not appear to be mediated by sleep duration (continuous), AIS score (continuous), DASH score (continuous), and physical activity (continuous) with all average causal mediation effects (ACMEs) crossed 0 (Table S3). In addition, in the RCS models, we further adjusted the major occupational hazards to the current participants (exposure to dust, heat stress, noise, and carbon monoxide), and the relationships remained comparable to Figure 2 (Supplementary Figure S1). Furthermore, when the BMI was further adjusted, different exposure metrics of night shift work (continuous) were still positively correlated with FMI and BF% (Supplementary Figure S2). Moreover, BMI ≥ 30 kg/m^2^ was also used to define obesity, and there was still no significant association between different exposure metrics of night shift work and obesity (Table S4). Subsequently, we analyzed the relationship between night shift work and obesity defined by BF% (≥35%) among nonobese-BMI participants (BMI < 28 kg/m^2^). After adjustment for confounders in Table 2, significantly elevated odds of obesity defined by BF% (≥35%) were also observed in the highest exposure categories of night shift work (Table S5). In order to evaluate whether the association of the duration of night shifts with FMI and BF% was generally impacted by decades of aging (Table S6), we adjusted the age group in GLM (other variables are the same as in Table 2), and the results were similar to those in Table 2.

## 4. Discussion

This cross-sectional study of female steelworkers provided novel insight into the effect of rotating night shift work on FMI and BF%. The present study also provided additional evidence concerning dose–response relationships of the duration of night shifts, cumulative number of night shifts, and cumulative length of night shifts with FMI and BF% among female steelworkers, which have never been reported in previous studies. In addition, we detected that the associations of different exposure metrics of night shift work with BF% and FMI were independent of BMI. For nonobese subjects by the BMI criterion (BMI < 28 kg/m^2^), the long duration and high frequency of night shift work were still associated with an increased risk of the excess accumulation of body fat.

Previous studies mostly focused on the relationship between shift work and BMI, while few studies focused on the effect of shift work on BF% or FMI. Apart from that, the relationship between shift work and increased BMI was inconsistent. Several methodological limitations may be responsible for the differences between studies. Several methodological limitations may be responsible for the differences between studies. First, many studies relied on self-reported weights and heights to calculate the BMI, which could have resulted in bias. Although studies have confirmed that weights measured by technicians and reported in questionnaires were highly correlated, the errors in different studies cannot be compared, especially when the conclusions were inconsistent [17,26]. In addition, the flexible implementation of night shift work, which differs across regions and employment sectors, poses a challenge to the exposure assessment in the field of shift work and health research.

Results of a previous study in this study setting showed a nonlinear relationship between duration of night shifts and overweight or obesity (BMI ≥ 24 kg/m^2^) among male steelworkers. However, the study did not explore the relationship between different exposure metrics of night shifts and BMI, and the conclusions between different studies were inconsistent [17,21]. Consistent with our findings, a previous small observational study that measured BF% among cleaning service employees concluded that compared with day workers, night workers had a greater body fat mass percentage despite similar BMI [15]. However, a study using principal component analysis (PCA) reported that the principal component (PC) of “body obesity component” (including body weight, BMI, BF%, WC, HC, WHR, WHtR, and other indicators) was not significantly associated with lifetime shift work (in years) among operators of iron ore extraction machinery, regardless of age adjustment [16]. As the principal components are linear combinations of the original variables, the principal components cannot completely replace the original variables, though the original variables are highly intercorrelated. Therefore, we cannot draw a direct relationship between duration of lifetime shift work and BF% based on this study. In addition, the absence of potential confounders such as environmental and behavioral factors may also lead to bias in that study. Interestingly, there was evidence that the levels of cardiometabolic risk factors (such as blood pressure, glucose, insulin, triglycerides, low density lipoprotein, fibrinogen, and CRP concentrations) were higher in nonobese (lean or overweight) subjects via the BMI criterion but obese via BF% compared with volunteers with normal body fat amounts, which suggests that BF% is a cardiometabolic risk factor independent of BMI and more sensitive than BMI [8]. This evidence may indirectly, at least in part, support the results of our study. As both shift work and BF% have been linked to the development of CVD [8,13,14], it seems logical to predict that elevated BF% could be a direct consequence of exposure to night shift work, thus serving as a potential mediator on the causal pathway between night shift work and CVD. Along this line, elevated FMI may also be a direct consequence of exposure to night shift work and thus act as a mediator of the association between night shift work and metabolic syndrome, as a large body of evidence suggests that both FMI and night shift work are closely related to metabolic syndrome [10,11,33,34,35]. In the RCS models, after BMI (continuous) was further adjusted, longer durations, cumulative numbers, and cumulative lengths of night shifts still increased subjects’ BF% and FMI. Likewise, among participants with BMI < 28 kg/m^2^, workers in the highest exposure categories of night shift work were still at significantly elevated odds of obesity defined by BF% (≥35%) compared with day workers. Therefore, night shift workers with a relatively “normal weight” should still be alert to the risk of body adiposity dysfunction, which may progress to CVD [6,36].

In terms of the association between shift work and BMI, the results were inconsistent. In our present study, after adjusting for potential confounders, no significant associations were found between different exposure metrics of night shift work and BMI. It should be noted that almost half of the studies on the relationship between night shift work and BMI or weight gain relied on self-reported weights and heights [17]. Although studies have confirmed that weights measured by technicians and reported in questionnaires were highly correlated, the errors between studies cannot be compared [17,26]. In fact, it was the excess accumulation of body fat that was responsible for most obesity-associated adverse health outcomes, as BMI cannot separate the lean mass from the fat mass [37]. Apart from that, coarse categorizations of shift work are commonly used to assign exposures in studies of shift work and obesity, although calls have been made to improve the quality of exposure assessment in this field [38]. Only a few studies have reported more detailed characteristics of night shift work and obesity (defined by BMI) [17,39]. Altogether, differences in study design, setting, exposure and outcome assessment, and population characteristics are thus more likely to be responsible for the discrepancy in the findings.

The potential mechanism linking night shift work with the excess accumulation of body fat may be the displacement of external behaviors and the misalignment of endogenous molecular circadian rhythms due to night shift work. Working night shifts means that an individual’s timing of the sleep/wake cycle is opposite to that of day workers, as well as the natural biological rhythm [40]. Peripheral appetite-regulating systems are modulated by the circadian rhythm and sleep/wake homeostasis through sympathetic and parasympathetic nervous activity and hypothalamic control of pituitary hormones [41]. Lower leptin levels and higher ghrelin levels have been linked to short sleep duration and night shift work in previous studies [42,43,44,45]. Therefore, the changes in appetite-stimulating profile mentioned above may contribute to the increased fat mass percentage in night shift workers [15]. In this study, we did find that the sleep duration of night shift workers was shorter than that of day workers. However, the results of causal mediation analysis showed that the associations of night shift work with FMI and BF% did not appear to be mediated by sleep duration and sleep quality, which suggests that the disruption of the sleep rhythm (the sleep/wake cycle), instead of sleep duration or sleep quality, may have a more fundamental effect on the circadian rhythm of total body adiposity [46]. Notably, the liver plays a crucial role in lipid metabolism. Indeed, liver lipid metabolism is regulated by the circadian clock through controlling the expression of circadian clock genes, enzymes that are critically involved in regulating various steps of lipid metabolism, and the regulation of lipid droplet dynamics [47,48]. Therefore, the disruption of the circadian rhythm due to rotating night shift work can directly lead to body adiposity dysfunction by affecting lipid metabolism in the liver. In addition, several behavioral lifestyles such as unhealthy dietary habits and low physical activity and environmental factors have been proposed as potential causes. It should be noted, however, that none of the mediation analyses found the dietary habits and physical activity to be statistically significant modifiers. After the DASH score, physical activity, bedroom ambient light level, and other potential confounders were adjusted, the association between night shift work and elevated body fat remained significant, thereby indicating that other as-yet-uncharacterized factors, such as feeding/fasting cycles, work family balance, and stress loads, might be involved [17]. Interestingly, animal studies have shown that the temporal regulation of feeding is a preventative intervention against obesity [49,50]. In addition, time-restricted feeding has emerged as an effective strategy for preventing sleep disturbances and mental health disorders [51]. Along this line, shift workers may be able to prevent the adverse effects of metabolic disorders by controlling their eating time during the active phase without altering caloric intake or nutrient composition [52].

The major strengths of our study include the detailed characteristics of exposure assessment, and potential confounders related to obesity. To our knowledge, this is the first study to explore the relationship between different exposure metrics of night shifts and body fat among female steelworkers. The present study also has some limitations. First, given the cross-sectional nature, we cannot infer the temporality of night shift work and body adiposity dysfunction. Second, chronotype was not assessed, which may have led to a confounding bias. Nonetheless, this may not be regarded as a major bias, as a previous well-designed study showed that chronotype was not a statistically significant modifier of the association between rotating night shift work and abdominal obesity [17]. Third, the “healthy worker effect” may result in an underestimation of the association between night shift work and body adiposity, if the reason for quitting night shift work was obesity-associated adverse health outcomes. Fourth, our survey population consisted of female steelworkers in north China, which limits our ability to generalize these results to the general population. Fifth, a fundamental limitation is that BIA is a predictive method that requires assumptions based on population mean values. An improved standardization of protocols for measurement is essential [53].

## 5. Conclusions

Altogether, a long duration and high frequency of night shift work may increase the accumulation of body fat in female steelworkers, and this effect is independent of BMI. Night shift workers with a relatively “normal weight” should still be alert to the risk of the excess accumulation of body fat, which is actually responsible for most obesity-associated adverse health consequences. Health interventions for related populations need to be improved, which is currently more focused on overall weight control. Future large-scale prospective studies are needed to confirm these findings and explore the cut-off point for obesity defined by FMI.

## Figures and Tables

**Table 1:** The general characteristics of study participants according to shift status.

Variables	Total	Day Work	Night Shift Work	
	*n* = 435	*n* = 91	*n* = 344	*p* Value
* Age (years), mean ± SD	44.1 ± 5.0	44.1 ± 5.0	44.1 ± 5.0	0.937 ^a^
Marital status, n (%)				0.329 ^b^
Married/Cohabitating	406 (93.3)	87 (95.6)	319 (92.7)	
Single/Divorced/Widow	29 (6.7)	4 (4.4)	25 (7.3)	
Smoking status, n (%)				0.036 ^b^
Nonsmoker	390 (89.7)	87 (95.6)	303 (88.1)	
Pre-/Current smoker	45 (10.3)	4 (4.4)	41 (11.9)	
Drinking status, n (%)				0.053 ^b^
Nondrinker	399 (91.7)	88 (96.7)	311 (90.4)	
Pre-/Current drinker	36 (8.3)	3 (3.3)	33 (9.6)	
Education level, n (%)				0.162 ^b^
High school or below	339 (77.9)	66 (72.5)	273 (79.4)	
University or college	96 (22.1)	25 (27.5)	71 (20.6)	
Ethnicity, n (%)				0.018 ^b^
Han	413 (94.4)	82 (90.1)	331 (96.2)	
Others	22 (5.1)	9 (9.9)	13 (3.8)	
Bedroom ambient light level, n (%)				0.603 ^b^
Darkest level	198 (45.5)	40 (44.0)	158 (45.9)	
Middle level	199 (45.8)	45 (49.5)	154 (44.8)	
Lightest level	38 (8.7)	6 (6.6)	32 (9.3)	
Physical activity (MET-h/week), median (IQR)	103.8 (82.9–126.9)	103.8 (84.5–131.7)	103.8 (78.9–125.8)	0.421^c^
DASH score, mean ± SD	22.9 ± 2.1	22.5 ± 2.1	23.0 ± 2.1	0.044 ^a^
Sedentary behavior (h), median (IQR)	4.0 (2.4–5.5)	3.0 (1.6–4.6)	4.3 (2.6–5.5)	<0.001 ^c^
Sleep duration (h), mean ± SD	6.8 ± 1.2	7.2 ± 1.2	6.7 ± 1.2	<0.001 ^a^
Insomnia, n (%)	154 (35.4)	33 (36.3)	121 (35.2)	0.847 ^b^
BMI (kg/m^2^), mean ± SD	23.8 ± 3.2	23.8 ± 3.1	23.8 ± 3.2	0.919^a^
WC (cm), mean ± SD	83.2 ± 11.0	83.7 ± 11.4	83.0 ± 11.0	0.571 ^a^
HC (cm), mean ± SD	98.8 ± 7.5	98.3 ± 7.7	99.1 ± 7.5	0.413 ^a^
WHR, mean ± SD	0.84 ± 0.07	0.85 ± 0.07	0.84 ± 0.07	0.111 ^a^
WHtR, mean ± SD	0.50 ± 0.06	0.50 ± 0.06	0.51 ± 0.06	0.069 ^a^
BF%, mean ± SD	29.1 ± 7.9	27.4 ± 7.5	29.6 ± 7.9	0.022 ^a^
FMI (kg/m^2^), mean ± SD	7.3 ± 2.7	6.8 ± 2.4	7.7 ± 2.8	0.039 ^a^
Menopausal status, n (%)				0.746 ^b^
Premenopausal	413 (94.9)	87 (95.6)	326 (94.8)	
Postmenopausal	22 (5.1)	4 (4.4)	18 (5.2)	
Current use of oral contraceptives, n (%)				0.225 ^b^
No	409 (94.0)	88 (96.7)	321 (93.3)	
Yes	26 (6.0)	3 (3.3)	23 (6.7)	

Values are expressed as the mean ± standard deviation (SD) or median (interquartile range (IQR)) or number (%); ^a^ *p*-values were from Student’s *t*-test; ^b^ *p*-values were from Pearson’s chi-square test; ^c^ *p*-values were from Wilcoxon Scores (Rank Sums). * The age ranges for the total participants, day workers, and night shift workers were 26–57, 31–52, and 26–57, respectively. BMI, body mass index; DASH, dietary approaches to stop hypertension; WC, waist circumference; HC, hip circumference; WHtR, waist-to-height ratio; WHR, waist-to-height ratio; BF%, body fat percentage; FMI, fat mass index.

**Table 2:** Associations of different exposure metrics of night shift work with BMI, BF%, and FMI from generalized linear models.

Exposure Metrics	BMI		FMI		PBF (%)	
	*β*	*p*	*β*	*p*	*β*	*p*
Duration of night shifts (years)						
Day work	0 (Ref)	0 (Ref)	0 (Ref)	0 (Ref)	0 (Ref)	0 (Ref)
Q1 (1–13)	−0.337	0.492	−0.051	0.904	0.103	0.932
Q2 (14–20)	−0.045	0.929	0.596	0.166	2.078	0.092
Q3 (21–26)	−0.427	0.378	0.496	0.235	1.494	0.211
Q4 (27–38)	−0.130	0.799	1.028	0.020 *	3.761	0.003 *
*p* trend		0.743		0.009 *		0.002 *
Cumulative number of night shifts (nights)						
Day work	0 (Ref)	0 (Ref)	0 (Ref)	0 (Ref)	0 (Ref)	0 (Ref)
Q1 (43–1157)	−0.380	0.437	-0.071	0.866	0.062	0.959
Q2 (1158–1790)	−0.033	0.947	0.590	0.167	2.118	0.083
Q3 (1791–2411)	−0.387	0.428	0.622	0.140	1.820	0.131
Q4 (2412–3580)	−0.152	0.768	0.928	0.038 *	3.440	0.007 *
*p* trend		0.783		0.011 *		0.003 *
Cumulative length of night shifts (hours)						
Day work	0 (Ref)	0 (Ref)	0 (Ref)	0 (Ref)	0 (Ref)	0 (Ref)
Q1 (344–9681)	−0.280	0.544	0.053	0.899	0.377	0.755
Q2 (9682–14600)	0.042	0.932	0.361	0.393	1.425	0.239
Q3 (14601–19941)	−0.540	0.274	0.708	0.097	2.479	0.043 *
Q4 (19942–42960)	−0.204	0.691	0.961	0.031 *	3.108	0.015 *
*p* trend		0.599		0.011 *		0.004 *
Average frequency of night shifts (nights/month)						
Day work	0 (Ref)	0 (Ref)	0 (Ref)	0 (Ref)	0 (Ref)	0 (Ref)
<3	−0.161	0.725	0.296	0.438	0.987	0.386
3–7	−0.113	0.841	0.778	0.093	2.043	0.146
>7	−0.343	0.426	0.862	0.013 *	2.252	0.036 *
*p* trend		0.429		0.007 *		0.030 *
Percentage of hours on night shifts						
Day work	0 (Ref)	0 (Ref)	0 (Ref)	0 (Ref)	0 (Ref)	0 (Ref)
<20%	−0.149	0.779	0.240	0.588	0.778	0.556
20–30%	−0.554	0.314	0.136	0.765	0.504	0.712
>30%	−0.175	0.675	0.916	0.006 *	2.464	0.018 *
*p* trend		0.670		0.038 *		0.013 *

* *p* < 0.05. Adjusted for age, smoking status, drinking status, education level, ethnicity, bedroom ambient light level, sleep duration, insomnia, sedentary behavior, DASH score, physical activity, marital status, menopausal status, and current use of oral contraceptives. The cut-off points of the average frequency of night shifts and percentage of hours on night shifts were chosen to secure a reasonable number of observations in each category.

**Table 3:** Associations of different exposure metrics of night shift work with obesity odds.

Exposure Metrics	Obesity-BMI		Obesity-BF%	
	OR (95% CI)		OR (95% CI)	
	Unadjusted	Adjusted	Unadjusted	Adjusted
Duration of night shifts (years)				
Day work	1.00	1.00	1.00	1.00
Q1 (1–13)	0.70 (0.24–2.06)	0.71 (0.22–2.24)	0.87 (0.34–2.22)	0.90 (0.34–2.39)
Q2 (14–20)	1.41 (0.55–3.60)	1.66 (0.58–4.70)	2.50 (1.12–5.58)	2.64 (1.12–6.22)
Q3 (21–26)	1.04 (0.39–2.75)	1.06 (0.37–3.04)	1.74 (0.77–3.97)	1.58 (0.67–3.74)
Q4 (27–38)	1.14 (0.44–2.95)	0.95 (0.32–2.84)	4.21 (1.97–9.02)	3.48 (1.50–8.08)
*p* trend	0.599	0.795	<0.001	0.002
Cumulative number of night shifts (nights)				
Day work	1.00	1.00	1.00	1.00
Q1 (43–1157)	0.68 (0.23–2.01)	0.69 (0.22–2.19)	0.96 (0.38–2.38)	0.99 (0.38–2.58)
Q2 (1158–1790)	1.34 (0.53–3.40)	1.60 (0.56–4.55)	2.35 (1.06–5.23)	2.53 (1.07–5.96)
Q3 (1791–2411)	1.20 (0.46–3.11)	1.15 (0.41–3.25)	2.06 (0.92–4.64)	1.80 (0.77–4.19)
Q4 (2412–3580)	1.07 (0.40–2.82)	0.89 (0.29–2.73)	3.90 (1.80–8.42)	3.11 (1.33–7.27)
*p* trend	0.578	0.786	<0.001	0.003
Cumulative length of night shifts (hours)				
Day work	1.00	1.00	1.00	1.00
Q1 (344–9681)	0.81 (0.29–2.27)	0.80 (0.26–2.44)	1.07 (0.44–2.61)	1.07 (0.42–2.71)
Q2 (9682–14600)	1.18 (0.46–3.07)	1.35 (0.47–3.82)	1.90 (0.84–4.29)	1.93 (0.81–4.56)
Q3 (14601–19941)	1.22 (0.47–3.15)	1.18 (0.41–3.37)	2.39 (1.07–5.31)	2.09 (0.90–4.86)
Q4 (19942–42960)	1.07 (0.40–2.82)	0.92 (0.30–2.83)	3.90 (1.80–8.42)	3.35 (1.43–7.81)
*p* trend	0.647	0.821	<0.001	0.001
Average frequency of night shifts (nights/month)				
Day work	1.00	1.00	1.00	1.00
<3	1.13 (0.45–2.81)	1.15 (0.44–3.03)	1.34 (0.60–3.04)	1.34 (0.56–3.14)
3–7	1.14 (0.38–3.40)	1.22 (0.37–4.05)	2.31 (0.95–5.60)	2.25 (0.87–5.81)
>7	1.01 (0.43–2.34)	0.94 (0.36–2.44)	2.78 (1.37–5.64)	2.50 (1.17–5.35)
*p* trend	0.935	0.784	0.001	0.008
Percentage of hours on night shifts				
Day work	1.00	1.00	1.00	1.00
<20%	0.81 (0.26–2.56)	0.81 (0.24–2.71)	1.26 (0.49–3.25)	1.23 (0.46–3.31)
20–30%	0.89 (0.28–2.82)	0.99 (0.29–3.38)	1.21 (0.46–3.23)	1.25 (0.45–3.47)
>30%	1.18 (0.53–2.62)	1.17 (0.47–2.92)	2.79 (1.40–5.59)	2.55 (1.21–5.39)
*p* trend	0.550	0.596	0.001	0.005

Adjusted for age, smoking status, drinking status, education level, ethnicity, bedroom ambient light level, sleep duration, insomnia, sedentary behavior, DASH score, physical activity, marital status, menopausal status, and current use of oral contraceptives. Obesity-BMI, BMI ≥ 28 kg/m^2^; Obese-BF%, BF% ≥ 35.0%.

## Data Availability

The data presented in this study are available on request from the corresponding author.

## Supplementary Materials

The following are available online at https://www.mdpi.com/article/10.3390/ijerph18126355/s1, Table S1: General characteristics of the included and excluded participants, Table S2: Partial Spearman correlation coefficients between different exposure metrics of night shift work and anthropometric measures, Table S3: Mediation analysis of potential mediators on the association of duration of night shifts with BMI, FMI, and BF%, Table S4: Association between different exposure metrics of night shift work and obesity odds (defined by BMI ≥ 30 kg/m^2^), Table S5: Association between different exposure metrics of night shift work and obesity odds (defined by BF% ≥ 35%) among nonobese-BMI participants (BMI < 28 kg/m^2^), Table S6: Associations of duration of night shifts with BF% and FMI from generalized linear models, Figure S1: Associations of duration of night shifts (continuous), cumulative number of night shifts (continuous), and cumulative length of night shifts (continuous) with BMI, FMI, and BF% from restricted cubic spline models after further adjusting for the major occupational hazards, Figure S2: Associations of duration of night shifts (continuous), cumulative number of night shifts (continuous), and cumulative length of night shifts (continuous) with BMI, FMI, and BF% from restricted cubic spline models after further adjusting for BMI.

## Abbreviations

BF%: body fat percentage; FMI: fat mass index; BMI: body mass index; CVD: cardiovascular disease; OR: odds ratio; CI: confidence interval; BIA: bioelectrical impedance analysis; DXA: dual-energy X-ray absorptiometry; LCCC: Lin’s concordance correlation coefficient; WHR: waist-to-hip ratio; WC: waist circumference; HC: hip circumference; WHtR: waist-to-height ratio; DASH: dietary approaches to stop hypertension; LAN: light at night; SD: standard deviation; IQR: interquartile range; GLM: generalized linear models; RCS: restricted cubic spline; AIS: Athens Insomnia Scale; ACME: average causal mediation effects.
