# Supplementary material for: Association of Rotating Night Shift Work with Body Fat Percentage and Fat Mass Index among Female Steelworkers in North China
Source: Int J Environ Res Public Health. 2021 Jun 11;18(12):6355. doi: 10.3390/ijerph18126355 (PMC8296160; doi:10.3390/ijerph18126355)
Supplement: Supplementary file 1 [file ijerph-18-06355-s001.zip › ijerph-1235193-supplementary.pdf]

Supplementary materials:
Association of rotating night shift work with body fat percentage and fat mass index among female steelworkers in North China

Shengkui Zhang [1], Han Wang [1], Yongbin Wang [2], Miao Yu [1] and Juxiang Yuan [1,*]

[1] Department of Epidemiology and Health Statistics, School of Public Health, North China University of Science and Technology, Tangshan, Hebei 063210, China; zhangsk@stu.ncst.edu.cn (S.Z.); wanghan@stu.ncst.edu.cn (H.W.); yumiao@stu.ncst.edu.cn (M.Y.)
[2] Department of Epidemiology and Health Statistics, School of Public Health, Xinxiang Medical University, Xinxiang, Henan 453003, China; 191035@xxmu.edu.cn
* Correspondence: yuanjxncst@163.com; Tel.: +86-0315-8805578

Assessment of occupational hazard factors

All related occupational hazard factors were measured by a qualified third-party company in accordance with the National Occupational Health Standards of the People's Republic of China (ICS 13.100). Exposure to dust was defined as workers who may be exposed to productive dust (inorganic dust, organic dust or mixed dust) during production (GBZ/T 229.1–2010) [1]. The total dust in the air of the workplace was collected at the breathing zone with a filter membrane, and its concentration was calculated based on the increased weight of the filter membrane and the amount of gas collected. When the dust concentration in the air was ≤50 mg/m^3, a filter membrane with a diameter of 37 mm or 40 mm was used; otherwise, a filter membrane with a diameter of 75 mm was used (GBZ/T 192.1–2007) [2]. Exposure to heat stress work was defined as the average wet-bulb globe temperature (WBGT) index of the workplace being equal to or greater than 25°C in the process of production (GBZ 2.2–2007) [3]. The WBGT index was measured by a black-wet bulb globe thermometer. If there was no productive heat source in the workplace, three measuring points were selected to take the average value of the WBGT index; if where there was a productive heat source, 3 to 5 measuring points were selected to take the average value of the WBGT index. If the workplace was isolated into different thermal or ventilated environments, 2 measuring points were selected to take the average value of the WBGT index (GBZ/T 189.7–2007) [4]. Exposure to industrial toxicants was defined as workers who may be exposed to a variety of harmful chemicals (the toxicant specifically refers to carbon monoxide in this population) during production (GBZ/T

229.2–2010) [5]. Carbon monoxide or carbon dioxide in the air of the workplace was pumped into a non-dispersive infrared-ray (NDIR) analyzer and selectively absorbed its infrared rays. The concentration of carbon monoxide was determined according to the absorption value (GBZ/T 160.28–2004) [6]. Exposure to noise was defined as workers who were exposed to a noisy environment where the 8-h/d or 40-h/week equivalent A-weighted sound pressure level was ≥80 dB, which may be harmful to health and hearing (GBZ/T 229.4–2012) [7]. The workplace production noise was measured by a sound level meter. If the distribution of the sound field in the workplace was uniform (between-field difference of A-sound levels were less than 3 dB(A)), three measuring points were selected to take the average value; otherwise, the workplace was divided into several sound level areas. In each sound field, two measuring points were selected to take the average value (GBZ/T 189.8–2007) [8].

Table of contents

Table S1 General characteristics of the included and excluded participants.

Table S2 Partial Spearman correlation coefficients between different exposure metrics of night shift work and anthropometric measures.

Table S3 Mediation analysis of potential mediators on the association of duration of night shifts with BMI, FMI, and BF%.

Table S4 Association between different exposure metrics of night shift work and obesity odds (defined by BMI≥30 kg/m^2).

Table S5 Association between different exposure metrics of night shift work and obesity odds (defined by BF%≥35%) among non-obese-BMI participants (BMI<28 kg/m^2).

Table S6 Associations of duration of night shifts with BF% and FMI from generalized liner models.

Figure of contents

Figure S1 Associations of duration of night shifts (continuous), cumulative number of night shifts (continuous), and cumulative length of night shifts (continuous) with BMI, FMI, and BF% from restricted cubic spline models after further adjusting for the major occupational hazards.

Figure S2 Associations of duration of night shifts (continuous), cumulative number of night shifts (continuous), and cumulative length of night shifts (continuous) with BMI, FMI, and BF% from restricted cubic spline models after further adjusting for BMI.

Table S1 General characteristics of the included and excluded participants.

Variables	Included	Excluded	
	n=435	n=151	*P* value
Age (years), mean ± SD	44.1 ± 5.0	45.1 ± 4.7	0.020
Marital status, n (%)			0.575
Married / Cohabitating	406 (93.3)	141 (94.6)	
Single / Divorced / Widow	29 (6.7)	8 (5.4)	
Smoking status, n (%)			<0.001
Non-smoker	390 (89.7)	118 (78.2)	
Pre-/Current smoker	45 (10.3)	33 (21.9)	
Drinking status, n (%)			<0.001
Non-drinker	399 (91.7)	124 (82.1)	
Pre-/Current drinker	36 (8.3)	27 (17.9)	
Education level, n (%)			0.072
Primary/Middle or high school	339 (77.9)	128 (84.8)	
University or college	96 (22.1)	23 (15.2)	
Ethnicity, n (%)			0.837
Han	413 (94.4)	144 (95.4)	
Others	22 (5.1)	7 (4.6)	
Bedroom ambient light level, n (%)			0.623
Darkest level	198 (45.5)	63 (41.7)	
Middle level	199 (45.8)	76 (50.3)	
Lightest level	38 (8.7)	12 (8.0)	
Sleep duration (h), mean ± SD	6.8 ± 1.2	6.7 ± 1.2	0.481
Insomnia, n (%)	154 (35.4)	61 (40.4)	0.273

Values are expressed as the mean ± SD or number (%); *P*-values were from Pearson's chi-square test for categorical variables and Student's t test or Wilcoxon Scores (Rank Sums) for continuous variables. IQR, interquartile range. The age ranges for the included and excluded participants were 26–57 and 26–55, respectively.

Table S2 Partial Spearman correlation coefficients between different exposure metrics of night shift work and anthropometric measures.

Anthropometric measures	Duration of night shifts		Cumulative number of night shifts		Cumulative length of night shifts	
	r	*p*	*r*	*p*	*r*	*p*
BMI	-0.01889	0.6988	-0.01719	0.7248	-0.02989	0.5403
FMI	0.11977	0.0138	0.10526	0.0306	0.11154	0.0219
PBF	0.15193	0.0017	0.13583	0.0052	0.14475	0.0029
WHR	-0.08061	0.0982	-0.08254	0.0904	-0.07717	0.1134
WHtR	0.03341	0.4937	0.02244	0.6457	0.02811	0.5647
WC	-0.01332	0.785	-0.02071	0.6714	-0.01717	0.7251

Values are expressed as partial Spearman correlation coefficients (r) and p values for significance test. Adjusted for age, smoking status, drinking status, education level, ethnicity, bedroom ambient light level, sleep duration, insomnia, sedentary behavior, DASH score, physical activity, marital status, menopausal status, and current use of oral contraceptives.

Table S3 Mediation analysis of potential mediators on the association of duration of night shifts with BMI, FMI, and BF%.

**Mediators**	**Outcome**	**Female**	
		**effect size**	**p-value**
Sleep duration (hours)	BMI		
	ACME	0.12 (-0.09 to 0.35)	0.27
	ADE	0.03 (-0.96 to 1.01)	0.98
	Total effect	0.09 (-0.79 to 1.19)	0.81
	Prop. Mediated	1.29 (-3.20 to 3.33)	0.83
Sleep duration (hours)	FMI		
	ACME	0.14 (-0.05 to 0.34)	0.13
	ADE	1.14 (0.40 to 2.02)	<0.01
	Total effect	1.28 (0.54 to 2.16)	<0.01
	Prop. Mediated	0.11 (-0.04 to 0.35)	0.13
Sleep duration (hours)	PBF		
	ACME	0.31 (-0.19 to 0.82)	0.22
	ADE	3.68 (1.54 to 6.19)	<0.01
	Total effect	3.98 (1.96 to 6.38)	<0.01
	Prop. Mediated	0.08 (-0.05 to 0.27)	0.22
AIS score	BMI		
	ACME	-0.01 (-0.01 to 0.05)	0.75
	ADE	0.10 (-0.78 to 1.20)	0.79
	Total effect	0.09 (-0.79 to 1.19)	0.81
	Prop. Mediated	-0.13 (-0.51 to 0.82)	0.96
AIS score	FMI		
	ACME	-0.01 (-0.08 to 0.04)	0.85
	ADE	1.29 (0.55 to 2.17)	<0.01

	Total effect	1.28 (0.54 to 2.16)	<0.01
	Prop. Mediated	-0.01 (-0.07 to 0.04)	0.85
AIS score	PBF		
	ACME	-0.02 (-0.23 to 0.12)	0.84
	ADE	4.00 (2.01 to 6.46)	<0.01
	Total effect	3.98 (1.96 to 6.38)	<0.01
	Prop. Mediated	-0.01 (-0.06 to 0.03)	0.84
DASH score	BMI		
	ACME	-0.04 (-0.20 to 0.06)	0.39
	ADE	0.10 (-0.78 to 1.20)	0.79
	Total effect	0.06 (-0.84 to 1.15)	0.87
	Prop. Mediated	-0.74 (-1.59 to 2.41)	0.96
DASH score	FMI		
	ACME	-0.03 (-0.15 to 0.07)	0.53
	ADE	1.29 (0.55 to 2.17)	<0.01
	Total effect	1.26 (0.51 to 2.11)	<0.01
	Prop. Mediated	-0.02 (-0.16 to 0.06)	0.53
DASH score	PBF		
	ACME	-0.07 (-0.41 to 0.20)	0.61
	ADE	4.00 (2.01 to 6.46)	<0.01
	Total effect	3.93 (1.96 to 6.33)	<0.01
	Prop. Mediated	-0.02 (-0.13 to 0.06)	0.61
Physical activity (MET-h/week)	BMI		
	ACME	0.09 (-0.04 to 0.25)	0.18
	ADE	0.10 (-0.78 to 1.20)	0.79
	Total effect	0.19 (-0.70 to 1.26)	0.67
	Prop. Mediated	0.46 (-3.26 to 2.24)	0.71
Physical activity (MET-h/week)	FMI		
	ACME	0.01 (-0.06 to 0.08)	0.76
	ADE	1.29 (0.55 to 2.17)	<0.01
	Total effect	1.30 (0.56 to 2.14)	<0.01
	Prop. Mediated	0.01 (-0.05 to 0.07)	0.76
Physical activity (MET-h/week)	PBF		
	ACME	-0.04 (-0.31 to 0.10)	0.71
	ADE	4.00 (2.01 to 6.46)	<0.01
	Total effect	3.96 (1.90 to 6.37)	<0.01
	Prop. Mediated	-0.01 (-0.10 to 0.03)	0.71

Adjusted for age, smoking status, drinking status, education level, ethnicity, bedroom ambient light level, sedentary behavior, marital status, menopausal status, and current estrogen use (mediator was included as continuous variable in each mediation analysis). DASH, dietary approaches to stop hypertension; AIS, Athens Insomnia Scale; BMI, body mass index; BF%, body fat percentage; FMI, fat mass index. ACME, average causal mediation effect; ADE, average direct effect.

Table S4 Association between different exposure metrics of night shift work and obesity odds (defined by BMI≥30 kg/m²).

Exposure metrics	Obesity-BMI (BMI ≥30 kg/m2)	
	Unadjusted OR (95% CI)	Adjusted OR (95% CI)
Duration of night shifts (years)		
Day work	1.00	1.00
Q1 (1–13)	0.53 (0.10–2.98)	0.54 (0.09–3.31)
Q2 (14–20)	1.41 (0.37–5.45)	1.65 (0.36–7.47)
Q3 (21–26)	1.04 (0.25–4.28)	0.97 (0.20–4.71)
Q4 (27–38)	2.12 (0.62–7.32)	1.66 (0.38–7.34)
P trend	0.144	0.350
Cumulative number of night shifts (nights)		
Day work	1.00	1.00
Q1 (43–1157)	0.52 (0.09–2.90)	0.53 (0.09–3.29)
Q2 (1158–1790)	1.34 (0.35–5.18)	1.55 (0.34–7.07)
Q3 (1791–2411)	1.06 (0.26–4.38)	0.97 (0.20–4.73)
Q4 (2412–3580)	2.23 (0.65–7.70)	1.80 (0.40–8.03)
P trend	0.117	0.300
Cumulative length of night shifts (hours)		
Day work	1.00	1.00
Q1 (344–9681)	0.52 (0.09–2.90)	0.52 (0.08–3.21)
Q2 (9682–14600)	1.33 (0.34–5.11)	1.57 (0.35–7.01)
Q3 (14601–19941)	1.07 (0.26–4.44)	0.99 (0.20–4.89)
Q4 (19942–42960)	2.23 (0.65–7.70)	1.73 (0.39–7.68)
P trend	0.115	0.295
Average frequency of night shifts (nights/month)		
Day work	1.00	1.00
<3	1.05 (0.27–4.02)	0.96 (0.23–4.06)
3–7	0.41 (0.05–3.77)	0.32 (0.03–3.49)
>7	1.68 (0.53–5.32)	1.69 (0.44–5.58)
P trend	0.302	0.326
Percentage of hours on night shifts		
Day work	1.00	1.00
<20%	0.36 (0.04–3.32)	0.32 (0.03–3.16)
20%–30%	0.81 (0.14–4.55)	0.79 (0.12–5.15)
>30%	1.65 (0.54–5.07)	1.76 (0.47–6.63)
P trend	0.176	0.167

Adjusted for age, smoking status, drinking status, education level, ethnicity, bedroom ambient light level, sleep duration, insomnia, sedentary behavior, DASH score, physical activity, marital status, menopausal status, and current use of oral contraceptives.

Table S5 Association between different exposure metrics of night shift work and obesity odds (defined by BF%≥35%) among non-obese-BMI participants (BMI<28 kg/m²)

Exposure metrics	Obese-PBF	
	Unadjusted OR (95% CI)	Adjusted OR (95% CI)
Duration of night shifts (years)		
Day work	1.00	1.00
Q1 (1–13)	1.06 (0.38–2.97)	1.17 (0.40–3.44)
Q2 (14–20)	1.88 (0.72–4.90)	2.09 (0.76–5.76)
Q3 (21–26)	1.50 (0.57–3.94)	1.40 (0.51–3.83)
Q4 (27–38)	4.21 (1.76–10.03)	3.51 (1.34–9.15)
P trend	0.001	0.002
Cumulative number of night shifts (nights)		
Day work	1.00	1.00
Q1 (43–1157)	1.17 (0.43–3.21)	1.30 (0.45–3.73)
Q2 (1158–1790)	1.76 (0.68–4.58)	1.99 (0.72–5.49)
Q3 (1791–2411)	1.73 (0.67–4.51)	1.57 (0.58–4.23)
Q4 (2412–3580)	3.94 (1.64–9.48)	3.17 (1.21–8.33)
P trend	0.001	0.003
Cumulative length of night shifts (hours)		
Day work	1.00	1.00
Q1 (344–9681)	1.19 (0.44–3.26)	1.29 (0.45–3.69)
Q2 (9682–14600)	1.38 (0.52–3.70)	1.47 (0.53–4.13)
Q3 (14601–19941)	2.12 (0.84–5.39)	1.94 (0.73–5.19)
Q4 (19942–42960)	3.94 (1.64–9.48)	3.41 (1.30–8.94)
P trend	0.001	0.001
Average frequency of night shifts (nights/month)		
Day work	1.00	1.00
<3	1.43 (0.56–3.65)	1.55 (0.59–4.07)
3–7	1.85 (0.65–5.30)	2.06 (0.69–6.19)
>7	2.53 (1.11–5.74)	2.57 (1.10–6.03)
P trend	0.013	0.020
Percentage of hours on night shifts		
Day work	1.00	1.00
<20%	1.32 (0.45–3.88)	1.43 (0.47–4.34)
20%–30%	1.47 (0.50–4.34)	1.60 (0.52–4.92)
>30%	2.44 (1.09–5.46)	2.52 (1.09–5.80)
P trend	0.015	0.018

Adjusted for age, smoking status, drinking status, education level, ethnicity, bedroom ambient light level, sleep duration, insomnia, sedentary behavior, DASH score, physical activity, marital status, menopausal status, and current use of oral contraceptives.

Table S6 Associations of duration of night shifts with BF% and FMI from generalized liner models.

Variable	FMI		PBF (%)	
	β	*p*	*β*	*p*
Duration of night shifts (years)				
Never	0 (Ref)	0 (Ref)	0 (Ref)	0 (Ref)
Q1 (1–13)	-0.132	0.754	-0.107	0.929
Q2 (14–20)	0.534	0.192	1.846	0.116
Q3 (21–26)	0.504	0.218	2.009	0.087
Q4 (27–38)	1.404	**0.006**	4.300	**0.003**
P trend		**0.008**		**0.003**

Adjusted for age group (20–29 years, 30–39 years, 40–49 years, 50–59 years), smoking status, drinking status, education level, ethnicity, bedroom ambient light level, sleep duration, insomnia, sedentary behavior, DASH score, physical activity, marital status, menopausal status, and current use of oral contraceptives.

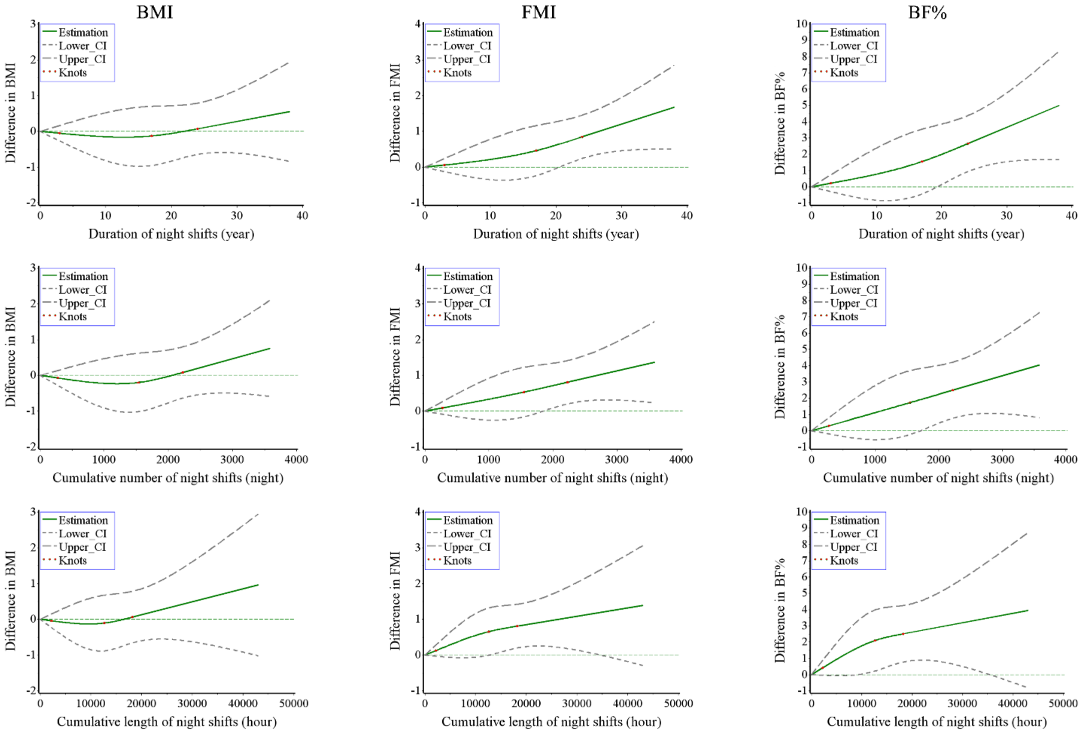

Figure S1 Associations of duration of night shifts (continuous), cumulative number of night shifts (continuous), and cumulative length of night shifts (continuous) with BMI, FMI, and BF% after further adjusted for major occupational hazards. The solid green lines, the long gray dashes and the short gray dashes represent the point estimate of the difference and the upper and lower limits of its 95% CI, respectively. Knots are represented by red dots. "Difference in BMI (FMI or BF%)", represents the difference in BMI (FMI or BF%) between night shift workers with any value of duration of night shifts (cumulative number or cumulative length of night shifts) with day workers. Adjusted for age, smoking status, drinking status, education level, ethnicity, bedroom ambient light level, sleep duration, insomnia, sedentary behavior, DASH score, physical activity, marital status, menopausal status, current use of oral contraceptives, dust exposure (No

or Yes), heat stress exposure (No or Yes), noise exposure (No or Yes), and carbon monoxide exposure (No or Yes). BMI, body mass index; BF%, body fat percentage; FMI, fat mass index; CI, confidence interval.

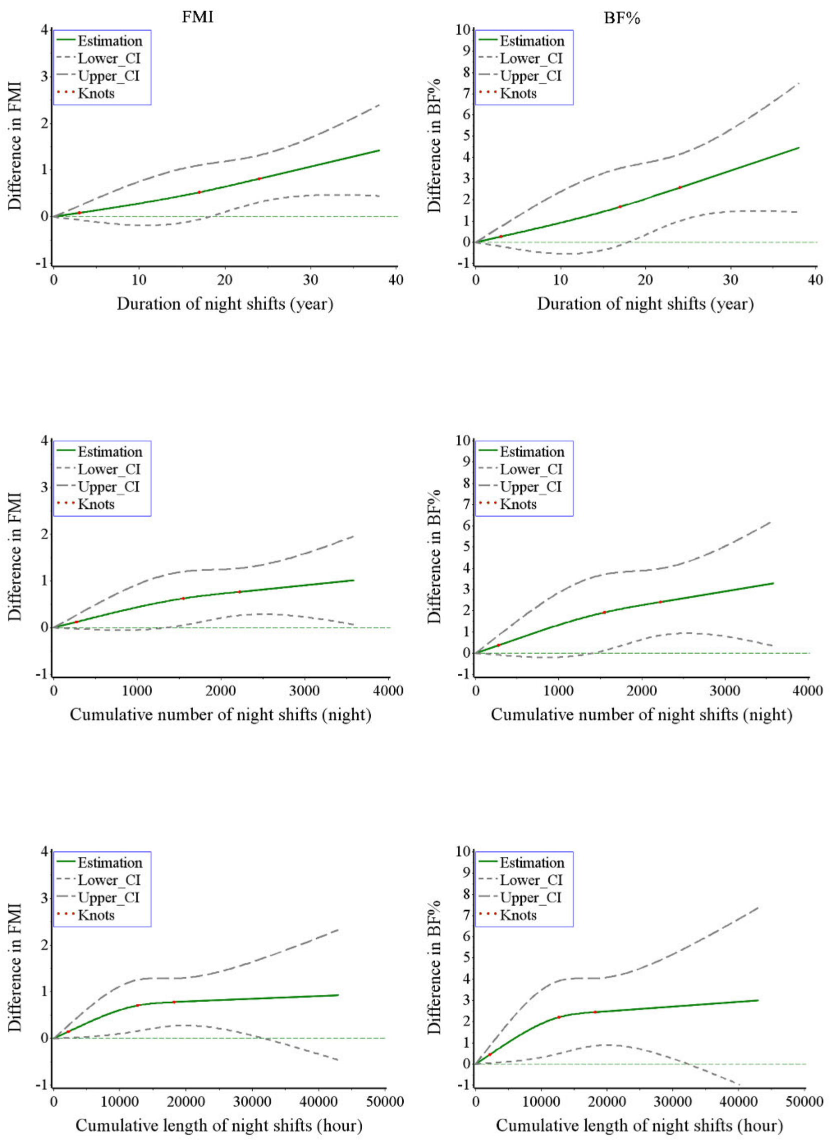

Figure S2 Associations of duration of night shifts (continuous), cumulative number of night shifts (continuous), and cumulative length of night shifts (continuous) with BMI, FMI, and BF% after further adjusted for BMI. The solid green lines, the long gray dashes and the short gray dashes represent the point estimate of the difference and the upper and lower limits of its 95% CI, respectively. Knots are represented by red dots. “Difference in BMI (FMI or BF%)”, represents the difference in BMI (FMI or BF%) between night shift workers with any value of duration of night shifts (cumulative number or cumulative length of night shifts) with day workers. Adjusted for age, smoking status, drinking status, education level, ethnicity, bedroom ambient light level,

sleep duration, insomnia, sedentary behavior, DASH score, physical activity, marital status, menopausal status, current use of oral contraceptives, and BMI (continuous). BMI, body mass index; BF%, body fat percentage; FMI, fat mass index; CI, confidence interval.

REFERENCES

1. GBZ/T GBZ/T 229.1–2010 Classification of occupational hazards at workplaces. Part 1: Occupational exposure to industrial dust. http://niohp.chinacdc.cn/zyysjk/zywsbzml/201210/t20121012_70490.htm
2. GBZ/T 192.1–2007 Determination of dust in the air of workplace. Part 1: Total dust concentration. http://niohp.chinacdc.cn/zyysjk/zywsbzml/201210/t20121012_70522.htm
3. GBZ 2.2–2007 Occupational exposure limits for hazardous agents in the workplace. Part 2: Physical agents. http://niohp.chinacdc.cn/zyysjk/zywsbzml/201303/t20130329_79199.htm
4. GBZ/T 189.7–2007 Measurement of physical agents in workplace. Part 7: Heat Stress. http://niohp.chinacdc.cn/zyysjk/zywsbzml/201210/t20121012_70527.htm
5. GBZ/T 229.2–2010 Classification of occupational hazards at workplaces. Part 2: Occupational exposure to chemicals. http://niohp.chinacdc.cn/zyysjk/zywsbzml/201210/t20121012_70489.htm
6. GBZ/T 160.28–2004 Methods for determination of inorganic carbon compounds in the air of workplace. http://niohp.chinacdc.cn/zyysjk/zywsbzml/201210/t20121015_70624.htm
7. GBZ/T 229.4–2012 Classification of occupational hazards at workplaces. Part 4: Occupational exposure to noise. http://niohp.chinacdc.cn/zyysjk/zywsbzml/201307/t20130715_84934.htm
8. GBZ/T 189.8–2007 Measurement of physical agents in workplace. Part 8: Noise. http://niohp.chinacdc.cn/zyysjk/zywsbzml/201210/t20121012_70526.htm
